# U-box ubiquitin ligase PPIL2 suppresses breast cancer invasion and metastasis by altering cell morphology and promoting SNAI1 ubiquitination and degradation

**DOI:** 10.1038/s41419-017-0094-4

**Published:** 2018-01-19

**Authors:** Zhaojun Jia, Miao Wang, Shujing Li, Xiahui Li, Xiao-Yan Bai, Zhaowei Xu, Yangyang Yang, Bowen Li, Yanan Li, Huijian Wu

**Affiliations:** School of Life Science and Biotechnology, Dalian University of Technology, Dalian, 116024 China

## Abstract

Metastasis is the leading cause of breast cancer fatalities. To develop new therapeutic strategies, the mechanisms underlying breast cancer invasion and metastasis need to be further investigated. Peptidylprolyl isomerase (cyclophilin)-like 2 (PPIL2) is a U-box-type E3 ubiquitin ligase belonging to the cyclophilin family. Proteins within this family are the major cytosolic binding proteins of the immunosuppressant drug cyclosporine A (CsA). Although PPIL2 has been reported to potentially be involved in cell migration, its role in breast cancer is still unclear. Herein, we demonstrate that PPIL2 suppressed metastasis in a breast cancer model by altering cell morphology and suppressing the epithelial–mesenchymal transition (EMT) process. Moreover, elevated PPIL2 inhibited EMT and breast cancer invasion by interacting with the classical EMT transcription factor, SNAI1, to enhance its ubiquitin-dependent degradation. Furthermore, PPIL2 protein level and stability was upregulated after CsA treatment, indicating that PPIL2 might be involved in CsA-mediated repression of EMT in breast cancer. Analysis of tissue samples taken from breast cancer patients showed a significant correlation between the expression of PPIL2 and the degree of cancer invasion and metastasis. In summary, these results would shed light on a potential clinical use of CsA in breast cancer patients.

## Introduction

Breast cancer is the most frequently diagnosed cancer in females worldwide, with a particularly high mortality rate^1–3^. Metastasis is the final stage of cancer progression where the carcinoma has progressed to a higher pathological grade of malignancy, and is therefore generally the leading cause of breast cancer-related death^4^. It is clear that the epithelial–mesenchymal transition (EMT), where cancer cells typically alter their shape and migrate through the extracellular matrix, is common in cancer invasion and metastasis. This change is typically accompanied by cell morphology remodeling, downregulation of homonymic adherens junction protein E-cadherin (*CDH1* gene), and upregulation of N-cadherin (*CDH2* gene)^5^ induced by SNAI1 (snail1), the predominant EMT-inducing transcriptional factor^6^. Interruption of metastasis pathways holds preclinical and clinical promise for breast cancer patients. Many pathways have been validated to interrupt metastasis, but have yet to be drugged. Novel antimetastatic mechanisms of postmarketing drug will enhance the efficacy of current treatments for breast cancer patients.

Cyclosporine A (CsA) is an immunosuppressant widely used to prevent the rejection of organ transplantations. CsA functions by binding intracellularly to the cyclophilin family proteins^7^. CsA inhibits breast cancer cell growth^8–10^, but the effects of CsA on the EMT process have been controversial. It has been known that CsA produces side effects like gingival hyperplasia and renal fibrosis by inducing type 1 EMT^11,12^. Berzal et al. observed that CsA enhanced SNAI1-induced EMT in renal tubular cells^13^. However, CsA inhibited cell migration and invasion in T47D cells^10^. In a case cohort of 21 439 female organ transplant patients being treated with CsA, treated patients had a lower risk than expected for de novo breast cancer, but a higher risk for skin cancer and non-Hodgkin’s lymphoma^14^. These results indicate a potential role of CsA in EMT and metastasis in breast cancer, but a potential usage of CsA in breast cancer treatment requires additional elucidation of mechanism of action, specifically of affected signaling pathways.

Peptidylprolyl isomerase (cyclophilin)-like 2 (PPIL2, also known as Cyp60 and CYP4) is a U-box-type E3 ubiquitin ligase belonging to the cyclophilin protein family, however, its biological function has not been clarified^15,16^. Recently, published data have shown that PPIL2 may play a role in cancer metastasis. Gaji et al. found that PPIL2 knockdown resulted in decreased deposition of F-actin, thereby affecting cell morphology and motility^17^. Meanwhile, PPIL2 had been found to decrease surface CD147 expression^18^. A recent report showed that the upregulation of CD147 promoted metastasis of cholangiocarcinoma by modulating the EMT process, indicating an effect of PPIL2 on EMT in cancer cells^19^. Furthermore, PPIL2 was found to be a target of miR-31 in a systematic analysis, which was also correlated with migration and invasion in many cancer types^20^. These findings suggest that PPIL2 might participate in cancer metastasis. Moreover, the role of PPIL2 in CsA-mediated EMT and metastasis warrants further investigation.

In the present study, we aimed to explore the role of PPIL2 in breast cancer metastasis. Revealing the precise mechanism of the role of PPIL2 in the EMT process might give insight into the potential use of CsA in breast cancer.

## Results

### PPIL2 alters cell morphology and suppresses metastasis in breast cancer cells

**Fig. 1:**
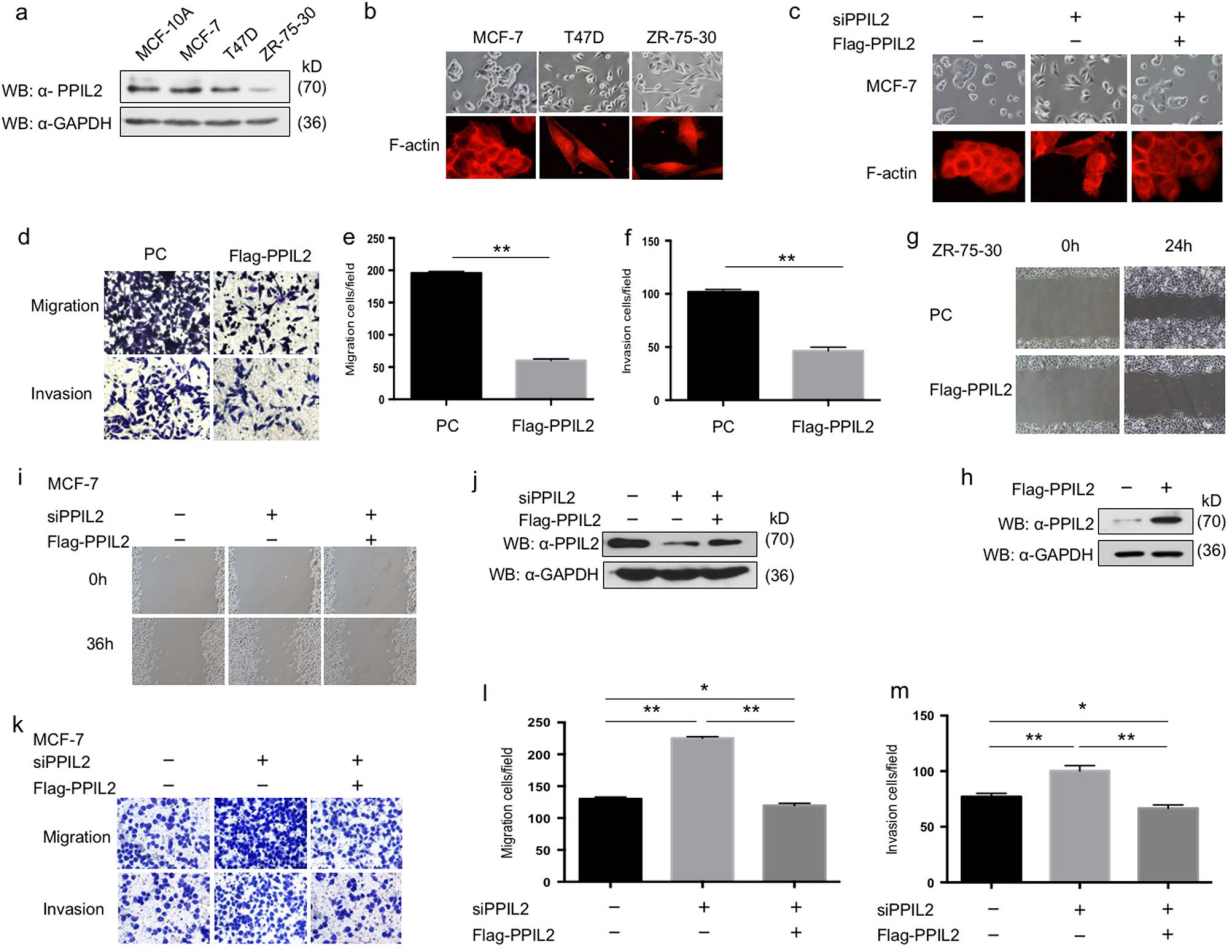
PPIL2 repressed metastasis in breast cancer cells **a** Western blot analysis of the expression of PPIL2 in MCF10A and three different breast cancer cells. **b** The morphology of MCF-7, T47D, and ZR-75-30 was checked by phase-contrast microscopic images (top, 20×), and F-actin of MCF-7, T47D, and ZR-75-30 was checked by immunofluorescence (bottom, 40×). **c** The changes of cell-to-cell junction were checked by phase-contrast microscopic images (top, 20×), and the F-actin deposition in MCF-7 cells with PPIL2 knockdown restored was checked by immunofluorescence (bottom, 40×) **d**–**f**, Transwell assay was used to assess the function of PPIL2 on migration and invasion in ZR-75-30 cells. Cell migration and invasion assays were performed in transwell chambers without or with Matrigel. Representative images from triplicate experiments are shown. The bar graphs show the number of migrating and invading cells for each category of cells. Each bar represents the mean ± S.D. from five independent experiments. ** means *P* < 0.01. **g** Scratch wound-healing assay assessing the effects of PPIL2 on the motility of ZR-75-30 cells (20×). **h** Western blot analysis was used to ensure the overexpression of PPIL2 in ZR-75-30 cells used for the Transwell assay and the scratch wound-healing assay in **d**–**g**. **i** Scratch wound-healing assay assessing the effects of PPIL2 knockdown on the motility of MCF-7 cells (10×). **j** Western blot analysis was used to confirm the level of PPIL2 in MCF-7 cells treated with siPPIL2 and Flag-PPIL2. Those cells were used in **c**, **I**, **k**–**m**. The siPPIL2 was designed to target the 3′UTR sequence of human *PPIL2* gene, Flag-PPIL2 that contained the exon of PPIL2 only. Thus, Flag-PPIL2 could be used as a siPPIL2-resistant isoform. **k**–**m** Transwell assay was used to evaluate the function of PPIL2 on migration and invasion in MCF-7 cells transfected with siPPIL2 and Flag-PPIL2. The bar graphs show the number of migrating and invading cells for each category of cells (right). **P < 0.01. *P < 0.05

To investigate the function of PPIL2 in breast cancer, the expression of PPIL2 was first measured in MCF10A, MCF-7, T47D, and ZR-75-30. The level of PPIL2 in MCF10A was comparable with epithelial MCF-7 cells. Both mesenchymal-like ZR-75-30 and T47D had lower PPIL2 expression compared to MCF-7 cells (Fig. 1a). MCF-7 cells were observed to form tight colonies and displayed a rounded epithelial morphology. Both mesenchymal-like ZR-75-30 and T47D cells scattered (Fig. 1b). The deposition of F-actin in these three cell lines was also measured using phalloidin staining. MCF-7 cells were observed to have mainly cytoplasmic F-actin, with strong cell-to-cell contact. Evident F-actin stress fibers were well organized and dispersed radially in the ZR-75-30 and T47D cells. Lamellipodia and filopodia were barely detected in MCF-7 cells (Fig. 1b). However, when PPIL2 expression was silenced in MCF-7 cells, cell shape was altered to resemble an irregular polygon, and cells had increased numbers of filopodia. The siPPIL2 was designed target to 3′UTR region. When transfected with Flag-PPIL2, which could be taken as siRNA-resistant PPIL2 isoform, the effect of siPPIL2 on cell morphology was restored (Fig. 1c, j).

We next investigated the function of PPIL2 in invasion and cell motility using transwell and wound-healing assay, respectively. The migration and invasion of ZR-75-30 cells were attenuated after Flag-PPIL2 transfection (Fig. 1d–h). The migration and invasion of MCF-7 cells were enhanced with PPIL2 knockdown (Fig. 1i–m). Taken together, our results show that PPIL2 changes cell morphology and represses cell migration and invasion in breast cancer cells.

### PPIL2 inhibits the EMT progress

**Fig. 2:**
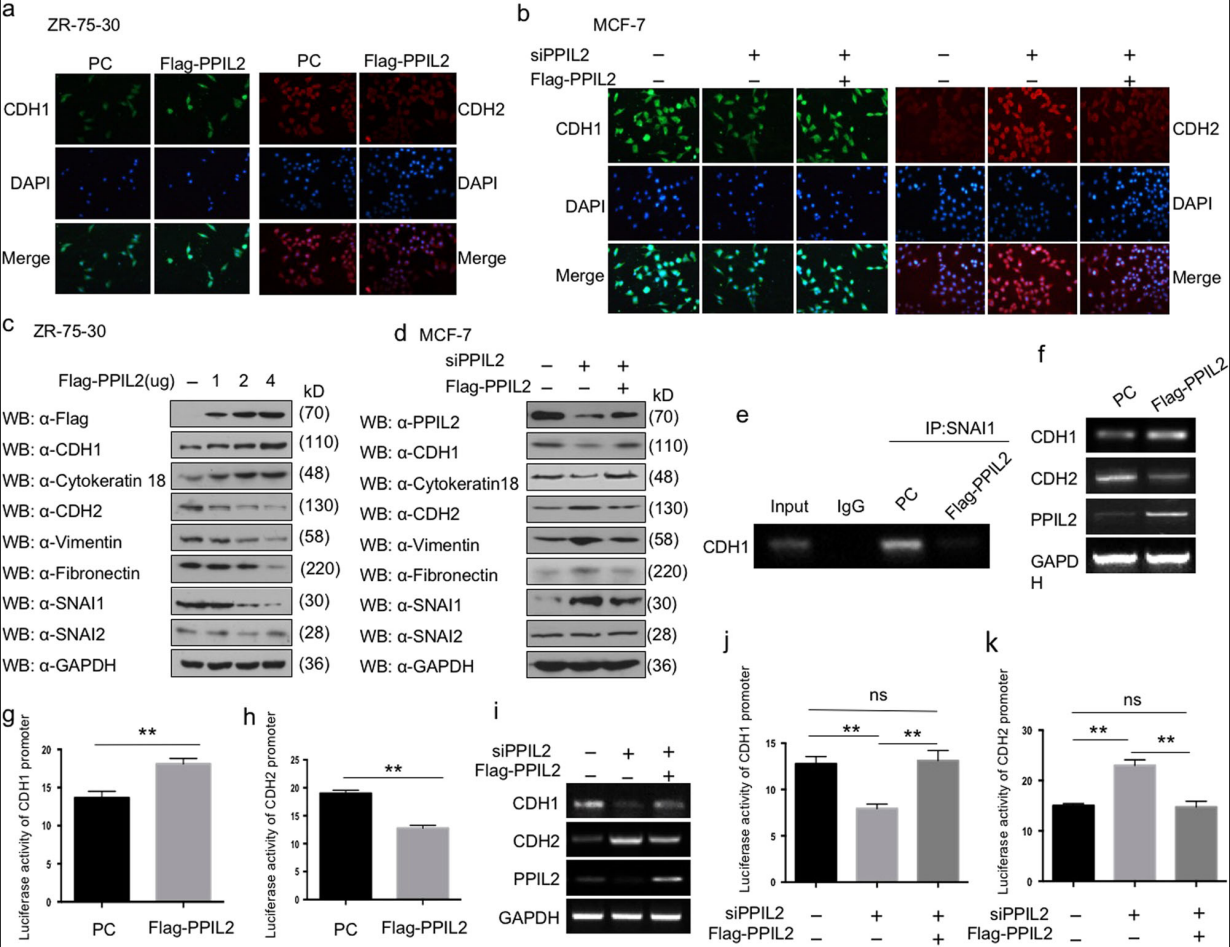
PPIL2 modulated the expression of EMT markers in breast cancer **a** The expression of CDH1 (E-cadherin) and CDH2 (N-cadherin) in ZR-75-30 cells that overexpressed Flag-PPIL2 was detected using immunofluorescence, and the level of PPIL2 was confirmed in Fig. 1h. **b** The expression of CDH1 and CDH2 in MCF-7 cell transfected with siPPIL2 and Flag-PPIL2 was detected using immunofluorescence, and the level of PPIL2 was confirmed in Fig. 1j. **c** Western blot was used to detect the level of EMT markers in ZR-75-30 cells overexpressed with PPIL2. CDH1 and cytokeratin 18 are epithelial EMT markers. CDH2, vimentin, and fibronectin are mesenchymal markers. **d** The protein level of EMT markers in MCF-7 cells was examined using western blot when PPIL2 was silenced or restored. **e** Chromatin immunoprecipitation assay showed the influence PPIL2 has on the binding of SNAI1 to the E-box of the CDH1 promoter in ZR-75-30 cells. **f** RT-PCR analysis showed the mRNA levels of CDH1 and CDH2 with PPIL2 overexpression in ZR-75-30 cells. **g**, **h** Reporter-gene assay showed the regulation of CDH1 and CDH2 luciferase reporter activity by PPIL2 overexpression in ZR-75-30 cells. Each bar represents the mean ± S.D. from five independent experiments. **P < 0.01. **i** RT-PCR analysis showed the mRNA levels of CDH1 and CDH2 with PPIL2 knockdown and restoration in MCF-7 cells. **j**, **k** Reporter-gene assay showed the regulation of CDH1 and CDH2 luciferase reporter activity by PPIL2 silencing and restoration in MCF-7 cells. Each bar represents the mean ± S.D. from five independent experiments. **P < 0.01. ns no significant difference

Since cancer metastasis usually accompanies the EMT process, we sought to investigate the role of PPIL2 in EMT by examining the expression of typical EMT markers. Immunofluorescence assay was used to detect the localization and expression levels of CDH1 and CDH2. In ZR-75-30 cells, PPIL2 overexpression increased the expression of CDH1, but decreased the expression of CDH2 (Fig. 2a). In contrast, depletion of PPIL2 in MCF-7 cells greatly reduced the expression of CDH1, but enhanced the expression of CDH2 (Fig. 2b).

Correspondingly, the protein levels of specific EMT markers were detected using western blot. We found that the expression of the epithelial cell-surface marker CDH1 and epithelial cytoskeletal marker cytokeratin 18 was elevated in PPIL2-overexpressing ZR-75-30 cells and reduced in PPIL2-knockdown MCF-7 cells. The levels of mesenchymal cell-surface marker CDH2 and mesenchymal cytoskeletal marker vimentin exhibited the opposite trend. Moreover, the level of mesenchymal extracellular marker fibronectin was reduced only when transfected with 4 μg of Flag-PPIL2 (Fig. 2c, d).

Since PPIL2 localizes mainly to the nucleus, we examined the effect of PPIL2 on SNAI1, which is the key regulator in EMT. Elevated PPIL2 suppressed the expression of SNAI1 in ZR-75-30 cells. When PPIL2 was silenced, the level of SNAI1 was increased in MCF-7 cells. Notably, a supplement of exogenous PPIL2 recovered the quantity of EMT markers and SNAI1. There were no distinct effects of PPIl2 on SNAI2 levels (Fig. 2d, e).

Considering that SNAI1 is the predominant transcription factor governing *CDH1* expression, a chromatin immunoprecipitation assay was used to investigate the influence PPIL2 has on the regulation of *CDHI* expression by SNAI1. The results show that PPIL2 reduces the recruitment of SNAI1 to the *CDH1* promoter (Fig. 2e). Furthermore, mRNA levels of the epithelial marker *CDH1* were found to be increased with PPIL2 overexpression, but the mRNA levels of the mesenchymal marker *CDH2* were found to be decreased (Fig. 2f). The reporter gene assay showed that the luciferase signal of *CDH1* was enhanced with PPIL2 overexpression, but the luciferase signal of *CDH2* was attenuated (Fig. 2g, h). An opposite change was observed in MCF-7 treated with siPPIL2 (Fig. 2i–k). Taken together, these results indicate that PPIL2 might have an important role in regulating EMT in breast cancer cells.

### PPIL2 interacts with SNAI1 through the U-box domain

**Fig. 3:**
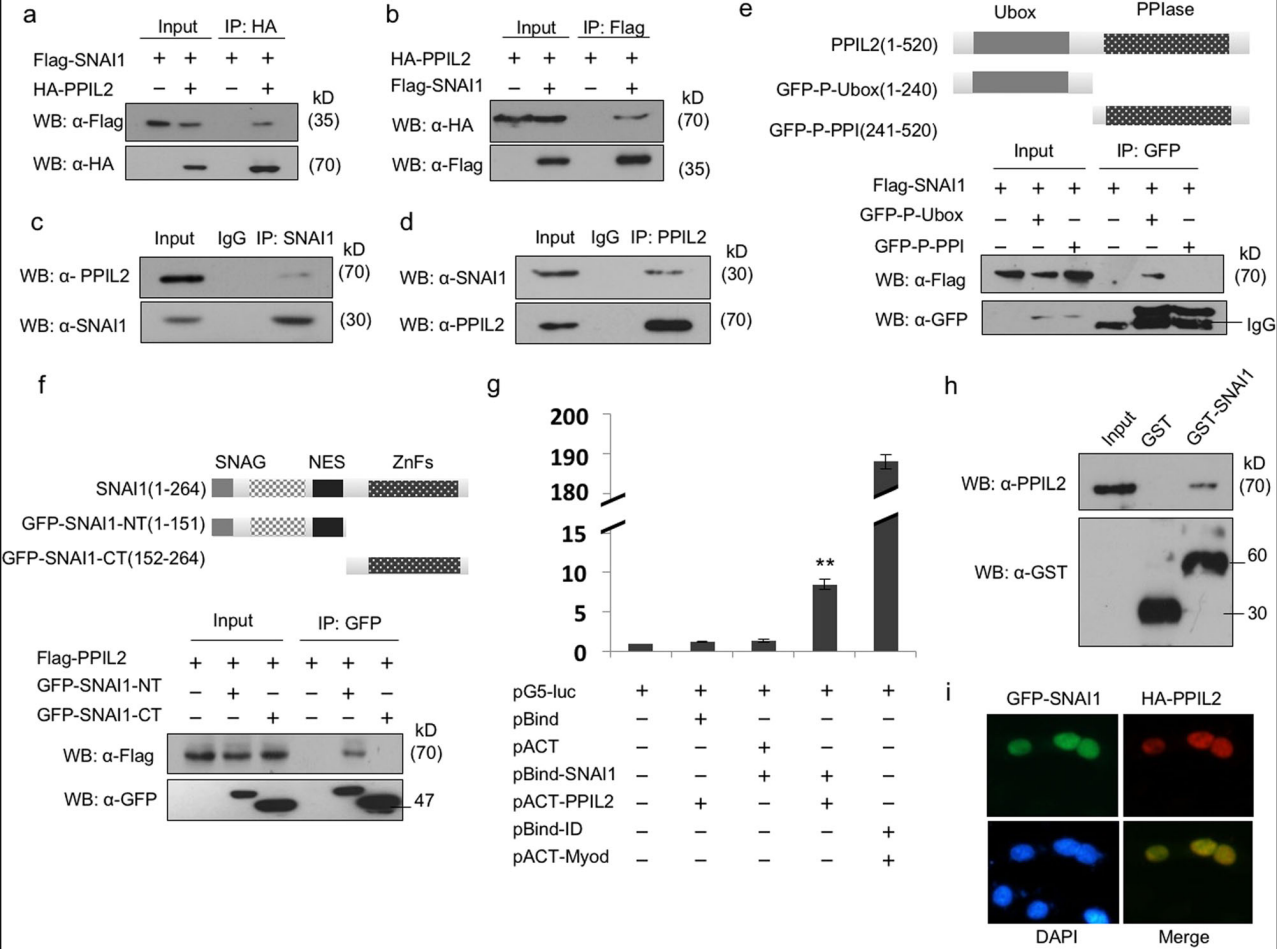
PPIL2 interacted with SNAI1 **a**, **b** Co-IP assay showed the interaction between exogenous PPIL2 and SNAI1 in T47D cells. **c**,**d** Co-IP assay showed the interaction between endogenous PPIL2 and SNAI1 in T47D cells. **e** Co-IP assay showed that PPIL2 interacted with SNAI1 via its U-box domain in T47D cells. **f** Co-IP assay showed that SNAI1 interacted with PPIL2 via its N terminal in T47D cells. **g** The direct interaction between PPIL2 and SNAI1 was observed using mammalian two-hybrid system. **h** GST pull-down assay showed the interaction between GST-SNAI1 and endogenous PPIL2 in MCF cells. **i** Immunofluorescence showed that PPIL2 (red) and SNAI1 (green) colocalized in the nucleus of MCF-7 cells (20×)

Since PPIL2 downregulates SNAI1 levels, we hypothesized that PPIL2 might inhibit EMT through this transcription factor. A co-immunoprecipitation (Co-IP) assay showed that overexpressed HA-PPIL2 interacts with Flag-SNAI1 in ZR-75-30 cells (Fig. 3a, b). The association between endogenous PPIL2 and SNAI1 was also demonstrated (Fig. 3c, d).

To define the region of PPIL2 that was involved in the interaction between PPIL2 and SNAI1, two truncated PPIL2 constructs with different PPIL2 domains were designed. The GFP-PPIL2-Ubox construct included only the U-box domain and GFP-PPIL2-PPI construct contained only the peptidylprolyl isomerases (PPI) domain. Only the U-box domain of PPIL2 interacted with SNAI1. Interestingly, overexpression of the GFP-PPIL2-Ubox construct, but not the GFP-PPIL2-PPI construct, inhibited SNAI1 expression (Fig. 3e). In addition, the binding regions of SNAI1 important for the interaction with PPIL2 were investigated by generating deletion mutants, GFP-SNAI1-NT (aa:1–151) and GFP-SNAI1-CT (aa:152–264). A Co-IP assay showed that PPIL2 interacted with the N terminus of SNAI1 (Fig. 3f).

In addition, the direct interaction between PPIL2 and SNAI1 was studied using the mammalian two-hybrid system (Fig. 3g). Glutathione S-transferase (GST) pull-down assays confirmed the direct interaction between GST-SNAI1 and endogenous PPIL2 (Fig. 3h). Furthermore, immunofluorescent staining revealed that PPIL2 and SNAI1 colocalized in the nucleus in MCF-7 cells (Fig. 3i). These results suggest that PPIL2 and SNAI interact.

### PPIL2 knockdown reduces ubiquitination of SNAI1

**Fig. 4:**
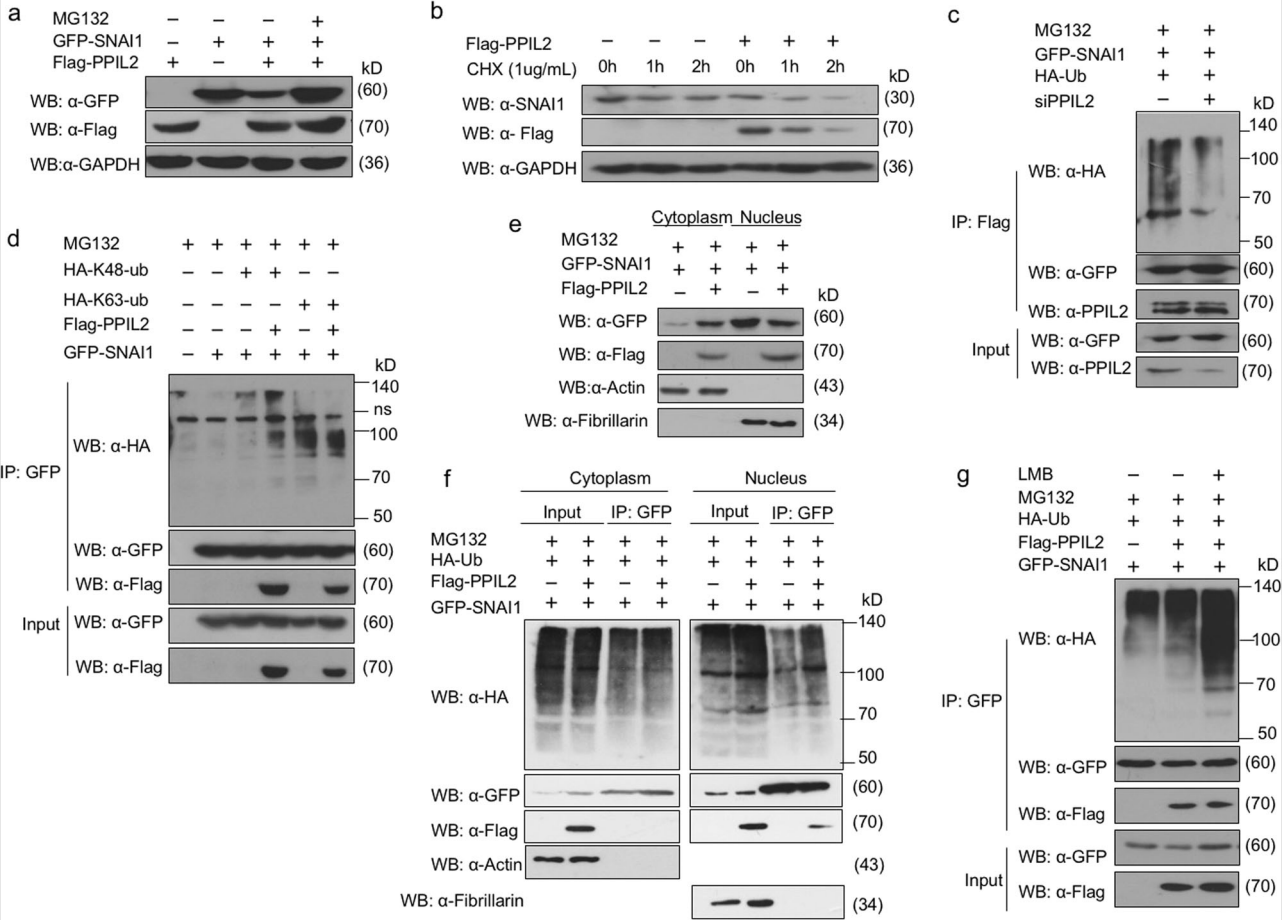
PPIL2 modulated SNAI1 stability and ubiquitination **a** Western blot assay showed that the decreased expression of SNAI1 in PPIL2-transfected ZR-75-30 cells was reversed 4 h after treatment with 10 μM MG132. **b** Western blot assay that showed overexpression of PPIL2 resulted in a shorter half-life of SNAI1 in ZR-75-30 cells treated with CHX. **c** Co-IP assay showed that the ubiquitin levels associated with SNAI1 were reduced when PPIL2 was silenced in MCF-7 cells. **d** Immunoblot analysis of lysates in 293T cells transfected for 24 h with GFP-SNAI1, along with HA-K63-Ub, HA-K48-Ub, and Flag-PPIL2, followed by immunoprecipitation with anti-GFP magnetic beads. **e** The cells were collected 24 h after transfection of Flag-PPIL2 and GFP-SNAI1, and the NE-PER™ Nuclear and Cytoplasmic Extraction Reagents (Themofisher, Beijing, China) were used to extract nuclear protein and cytoplasmic protein. Western blot assay showed that elevated PPIL2 reduced nucleus location of SNAI1 in ZR-75-30 cells. **f** Cytoplasmic protein and nuclear protein were extracted for separate immunoprecipitation. The results showed that PPIL2 increased the ubiquitination of SNAI in the nucleus but had no effect on the ubiquitination of SNAI1 in the cytoplasm. Western blot assay showed that elevated PPIL2 increased the enrichment of SNAI1 ubiquitination in the nuclear but not the cytoplasmic fractions. **g** Co-IP assay showed that PPIL2 prompted the ubiquintination of SNAI1 in the nucleus in ZR-75-30 cells after incubation with 5 ng/ml Leptomycin B for 3 h

We further defined the mechanism by which PPIL2 regulates SNAI1. The decreased expression of SNAI1 in PPIL2-transfected ZR-75-30 cells was reversed 4 h after treatment with the proteasome inhibitor MG132 (Fig. 4a). Expression of PPIL2 resulted in a shorter half-life of SNAI1 in ZR-75-30 cells, as assessed by treatment with a protein synthesis inhibitor cycloheximide (Fig. 4b).

As PPIL2 is a U-box-type ubiquitin E3 ligase, we investigated its role in regulating the ubiquitination of SNAI1. A predictable drop in ubiquitin levels associated with SNAI1 was observed when PPIL2 was silenced with a small interfering RNA (Fig. 4c). HA-K48-Ub and HA-K63-Ub plasmids were used to identify the ubiquitination type of SNAI1 induced by PPIL2^21^. HA-K48-Ub is a ubiquitin plasmid with all lysines mutated except K48. HA-K63-Ub is a ubiquitin plasmid with all lysines mutated except K63. Elevated PPIL2 levels mainly induced the accumulation of K48-linked polyubiquitylation of SNAI1, while the level K63-linked polyubiquitylation of SNAI1 was weakly reduced. (Fig. 4d). PPIL2 and SNAI1 colocalized in the nucleus. Next, we verified the effect of PPIL2 within subcellular compartments. We transfected the 293T cells with GFP-SNAI1 or GPF-SNAI1 plus Flag-PPIL2. After the cells were collected, cytoplasmic protein and nuclear protein were extracted for separate immunoprecipitation. The results showed that PPIL2 increased the ubiquitination of SNAI in the nucleus but had no effect on the ubiquitination of SNAI1 in the cytoplasm. It was found that elevated PPIL2 expression reduced nucleus localization of SNAI1 (Fig. 4e) and increased the enrichment of ubiquitin chains in the nuclear but not the cytoplasmic fractions (Fig. 4f). A similar pattern of accumulation of SNAI1 ubiquitination in the nucleus was observed in ZR-75-30 cells incubated with Leptomycin B, a nuclear export inhibitor (Fig. 4g). These results indicate that PPIL2 might modify SNAI1 degradation and ubiquitination in the nucleus.

### PPIL2 is involved in CsA-regulated breast cancer metastasis

**Fig. 5:**
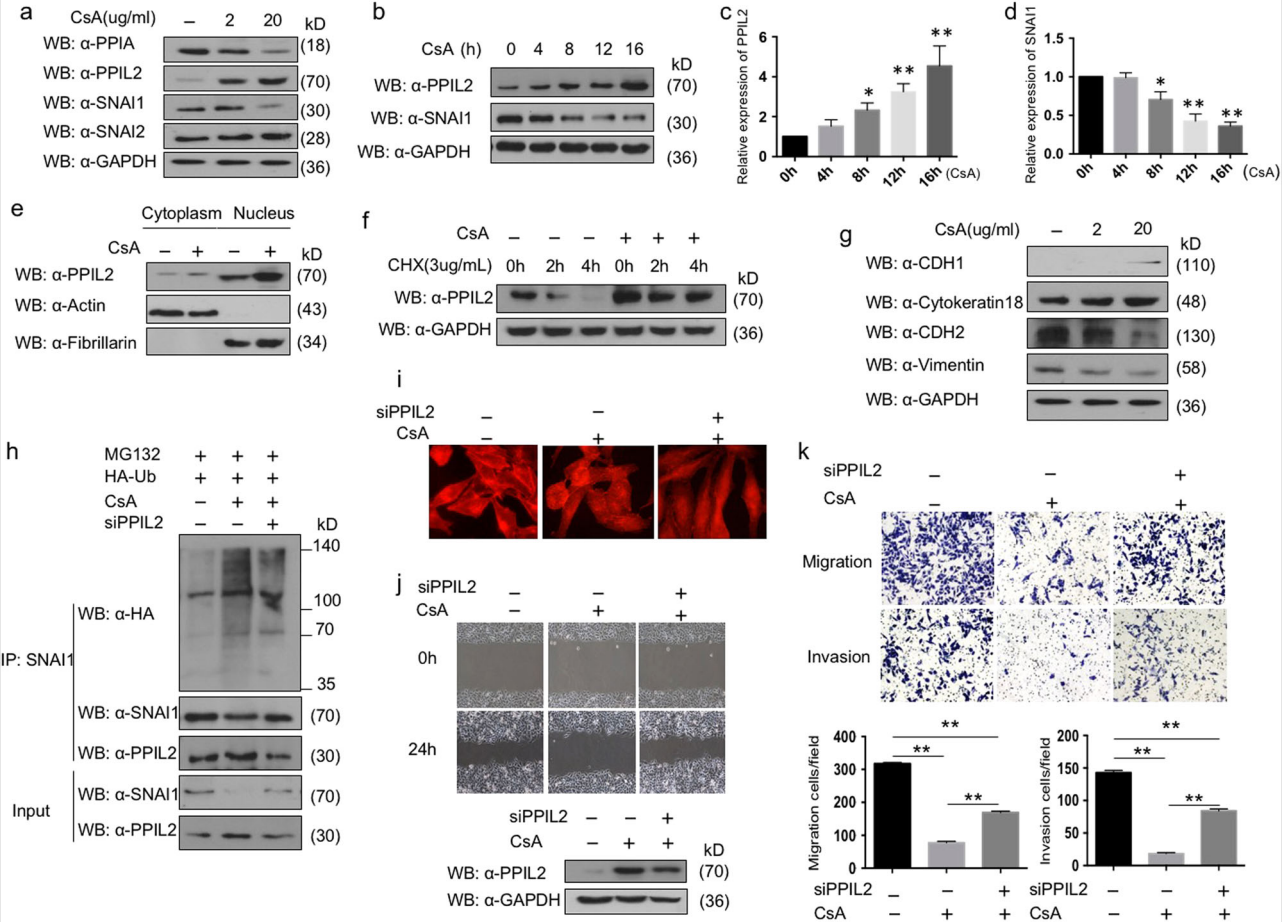
The effect of CsA on PPIL2 and EMT in breast cancer cells **a** Concentrations of 2 and 20 μg/ml CsA were added into the medium 16 h after the ZR-75-30 cells were plated. DMSO was taken as negative control. The cells were collected 12 h later and prepared for western blot. The expression of PPIL2, SNAI1, and SNAI2 was detected. **b**–**d** A concentration of 20 μg/ml CsA was added 16 h after the ZR-75-30 cells were plated. The cell lysate was collected at 4, 8, 12, and 16 h, respectively. The result of western blot showed that PPIL2 level increased in a time-dependent manner with CsA treatment within 16 h. The bar graphs showed the relative level of PPIL2 and SNAI1 in three independent experiments. *P < 0.05, **P < 0.01 **e** CsA raised PPIL2 abundance both in the cytoplasm and the nucleus in ZR-75-30 cells. **f** CsA prolonged the half-period of PPIL2 in ZR-75-30 cells. **g** The expression of EMT markers was examined in ZR-75-30 cells treated with 2 and 20 μg/ml CsA. **h** The level of SNAI1 ubiquitination was upregulated with CsA treatment, but PPIL2 silence attenuated the effect of CsA. **i** CsA induced F-actin arrangement in ZR-75-30 cells. Segments of F-actin fiber evenly distributed throughout the cell. The fibrous construction was reconstructed and prolonged after PPIL2 was silenced (40×). **j**, **k** the migratory and invasive ability of ZR-75-30 cells treated with 20 μg/ml was dampened compared to cells treated with DMSO (20×). The effect of CsA on cell motility was negated with siPPIL2 transfection. The bar graphs show the number of migrating and invading cells for each category of cells. Each bar represents the mean ± S.D. from three independent experiments. **P < 0.01

The cyclophilin protein family is the target of the natural product CsA. However, there has been no evidence of CsA binding to PPIL2 in an isothermal calorimetry assay^22^. But the expression of PPIL2 increased in ZR-75-30 cells treated with 2 and 20 μg/ml of CsA, compared to dimethyl sulfoxide (DMSO) treatment. The concentration level of peptidylprolyl isomerase A (PPIA, also known as cyclophilin A, CypA) was lowered as a positive control. Meanwhile, CsA treatment repressed SNAI1 level. The effect of CsA on SNAI2 was minute (Fig. 5a). The pharmacokinetic parameters of cyclosporine are known to show large intraindividual and interindividual variability^23^. The results of multiple independent experiments showed that PPIL2 levels increased in a time-dependent manner with CsA treatment within 16 h. Obvious accumulation of PPIL2 was detected 8 h after CsA treatment (Fig. 5b–d). Meanwhile, SNAI1 protein decreased along with CsA treatment throughout the time course. Furthermore, we noticed that CsA elevated PPIL2 levels in both the cytoplasm and nucleus (Fig. 5e). When treated with cycloheximide, the stability of PPIL2 increased (Fig. 5f). In conclusion, CsA induced PPIL2 accumulation in breast cancer cells.

Since CsA depressed SNAI1, we verified the function of CsA in EMT. The expression of EMT markers was examined in ZR-75-30 cells treated with 2 and 20 μg/ml CsA, respectively. CDH1 levels increased with CsA treatment, but there was little effect of CsA on cytokeratin 18 levels. Conversely, the expression of mesenchymal markers CDH2 and vimentin was decreased after CsA treatment (Fig. 5g). These results indicate that CsA could repress EMT in breast cancer cells.

To clarify the role of PPIL2 in CsA-induced repression of SNAI1, the level of SNAI1 ubiquitination was tested. CsA induced the ubiquitination of SNAI1, but the effect was attenuated after PPIL2 was silenced. (Fig. 5h). Moreover, the distribution of F-actin was also measured in ZR-75-30 cells treated with CsA. After treatment with CsA for 24 h, evenly distributed segments of F-actin fibers throughout the cell were observed. On the contrary, the fibrous pattern was altered and prolonged with CsA after PPIL2 was silenced (Fig. 5i). Next, we explored the antimetastatic function of CsA. The wound healing and transwell assays showed that the migratory and invasive ability of ZR-75-30 cells treated with 20 μg/ml was dampened compared to cells treated with DMSO. However, the effect of CsA on cell motility was negated with siPPIL2 transfection (Fig. 5j, k). Collectively, these findings are consistent with the speculation that PPIL2 plays a partial role in the antimetastasis mechanism of CsA in breast cancer cells.

### PPIL2 inhibits breast cancer metastasis in vivo

**Fig. 6:**
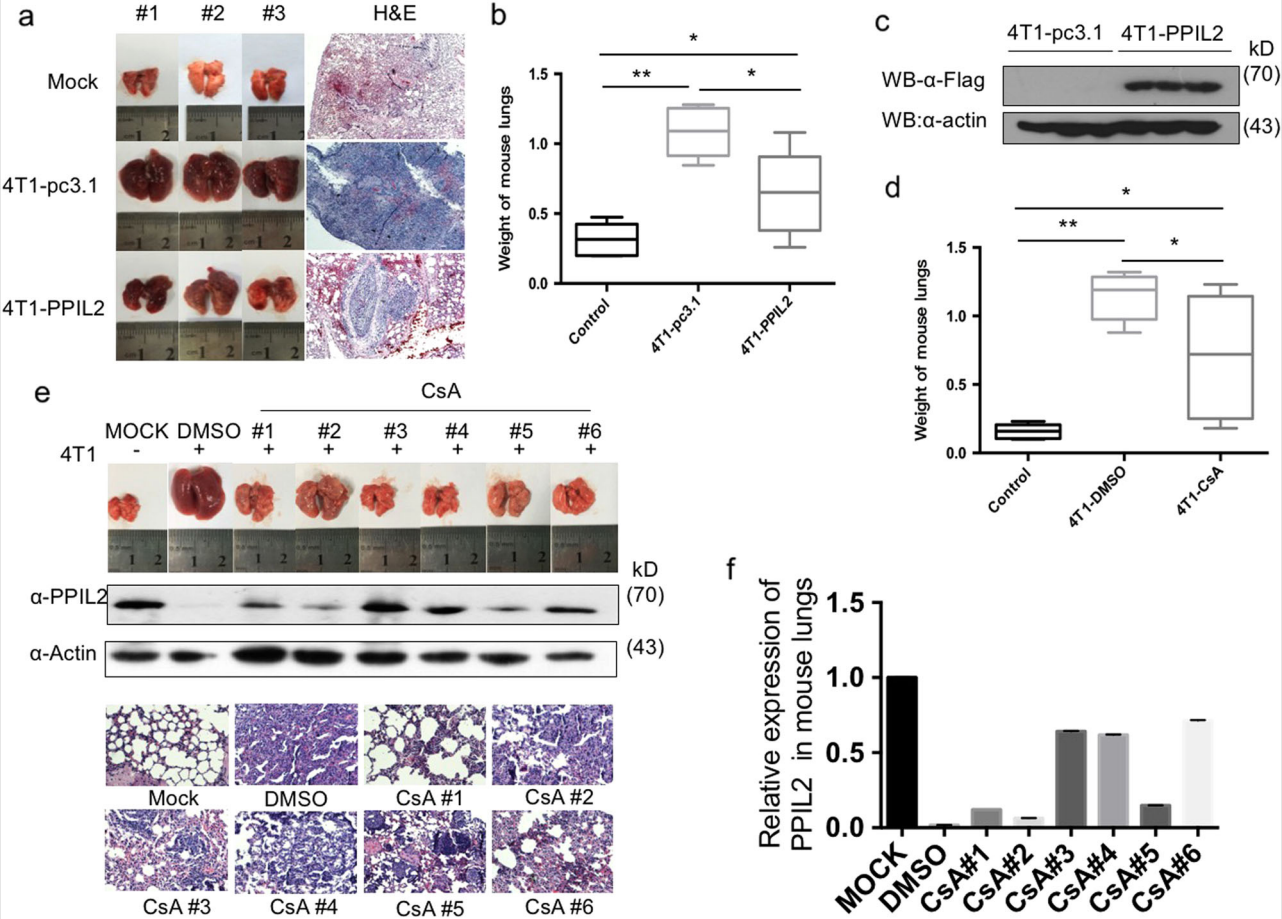
PPIL2 inhibited metastasis in vivo **a** Mice injected with PPIL2-overexpressing 4T1/Luc cells showed a significant reduction of lung metastases compared to injection with 4T1-pc3.1 cells. *n* = 5 mice per group. **b** The weight of lungs in the 4T1-PPIL2-injected group was also significantly decreased compared to the 4T1-pc3.1-injected group. Each bar represents the mean ± S.D. from five independent experiments. *P < 0.05. **P < 0.01. **c** The level of Flag-PPIL2 in lung tissue was examined to ensure that 4T1/Luc cells had migrated to the lung. **d** Mice injected with CsA (25 mg/kg/day, *n* = 8) showed a significant reduction of lung metastases compared to injection with equal volume of 10% DMSO (*n* = 5). Each bar represents the mean ± S.D. from five independent experiments. *P < 0.05. **P < 0.01. **e** The lung metastases and endogenous PPIL2 expression in the mice injected with CsA in mouse lung tissue. The lungs tissue slices were observed after hematoxylin–eosin staining (4×). **f** The bar graphs show the quantitative expression of PPIL2 in **e**

To study the role of PPIL2 in metastasis in vivo, an animal metastasis model was used, where BALB/c mice were injected with 4T1/Luc cells, and a mouse breast cancer cell line expression luciferase. We found that mice injected with PPIL2-overexpressing 4T1/Luc cells showed a significant reduction of lung metastases compared to injection with 4T1-pc3.1 cells. However, injection with 4T1-pc3.1 cells had more metastatic loci than the MOCK group (Fig. 6a). The weight of lungs in the 4T1-PPIL2-injected group was also significantly decreased compared to the 4T1-pc3.1-injected group (Fig. 6b). The level of Flag-PPIL2 in lung tissue was examined to ensure that 4T1/Luc cells had migrated to the lung (Fig. 6c). These results demonstrate that PPIL2 represses breast cancer metastasis in an animal metastasis model.

We also investigated the effect of CsA on metastasis in mice. A significant drop in the weight of lungs from the CsA-treated group compared to the DMSO group was observed. There were no significant differences in weights of lungs between the CsA group and the MOCK group (Fig. 6d). We then chose five lungs with less metastatic carcinoma from the CsA group for PPIL2-level measurements. We noted that higher PPIL2 expression was correlated with fewer lung metastases in the CsA-treated group. Metastatic status was confirmed using hematoxylin–eosin staining (Fig. 6e, f). Taken together, these data support the hypothesis that PPIL2 is involved in breast cancer metastasis in our animal model.

**Fig. 7:**
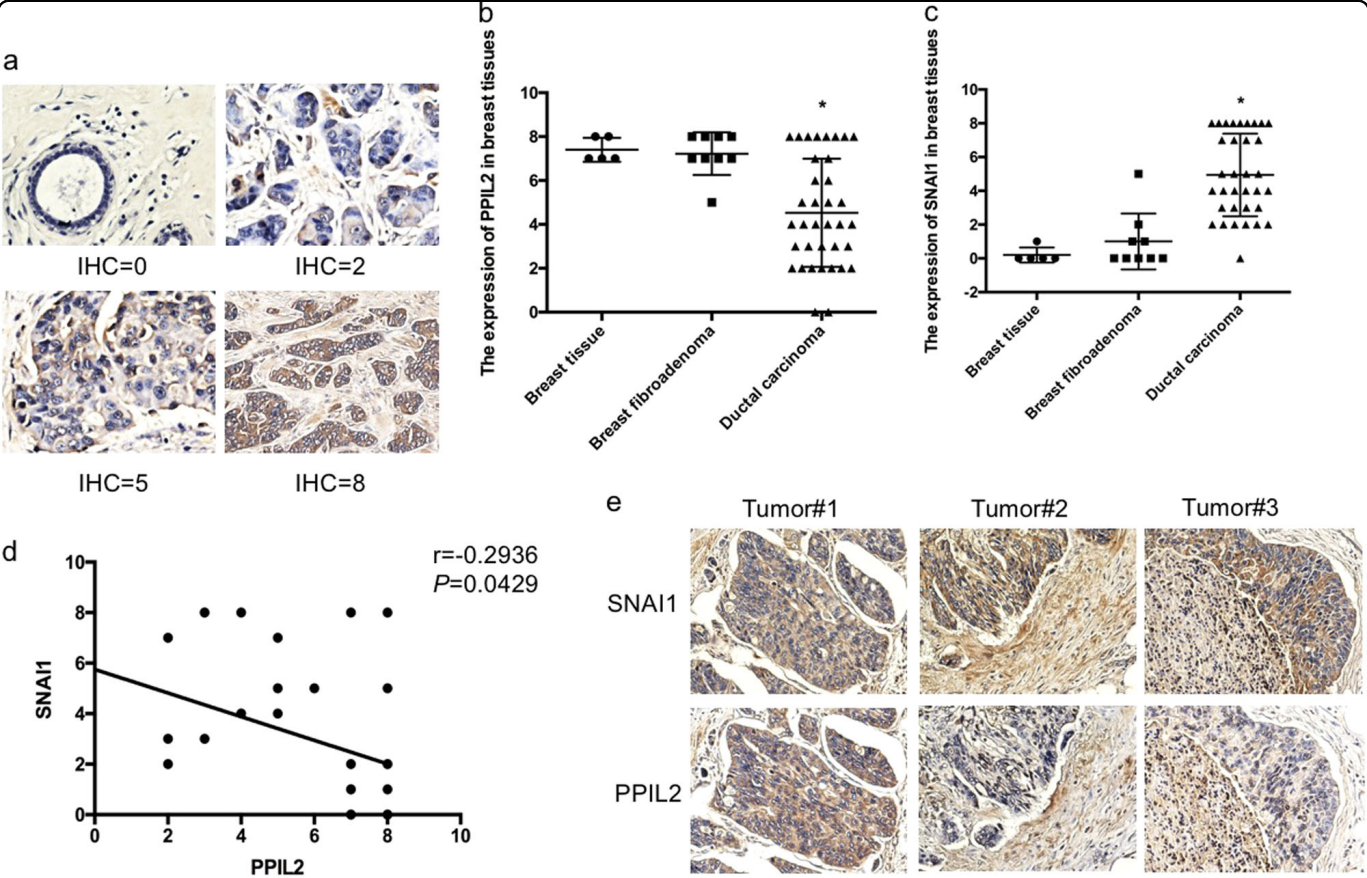
The expression of PPIL2 in clinical cases **a** A IHC score was assigned to the expression level of PPIL2 and SNAI1 in normal/pericarcinomatous breast tissues (*n* = 5), breast fibroadenoma tissues (*n* = 9), and ductal breast cancer tissues (*n* = 34, 20×). **b**, **c** The figures showed that the expression of PPIL2 was significantly less in ductal breast cancer tissues. The expression of SNAI1 was higher in ductal breast cancer tissues than the others. *P < 0.05. **d** There was a negative correlation between the expression of PPIL2 and SNAI1 level in different breast tissues (*r* = −0.2936 *P* = 0.0429). **e** Lower PPIL2 levels were observed in areas where cancer cells appeared to be traversing the basement membrane to the peripheral tissue (10×)

To determine the clinical relevance of our in vitro observations, we analyzed the expression levels of PPIL2 by immunohistochemistry (IHC) in human breast tissue samples including five normal/pericarcinomatous samples, nine fibroadenoma samples, and 34 ductal carcinoma samples. Based on the IHC scoring system defined in Materials and Methods (Fig. 7a), PPIL2 expression was lower in ductal breast carcinoma tissue compared to normal/pericarcinomatous and fibroadenoma breast tissue. There were no significant differences in PPIL2 expression between breast tissue and breast fibroadenoma tissue (Fig. 7b). Conversely, SNAI1 was found to be elevated in breast ductal carcinoma tissue compared to the other tissue types (Fig. 7c). In addition, the SNAI1 expression was negatively correlated with PPIL2 levels (*r* = −0.2936, *P* = 0.0429, 95% confidence interval = (−0.5333, −0.01021), Fig. 7d).

Interestingly, we observed lower PPIL2 levels in areas where cancer cells appeared to be traversing the basement membrane to the peripheral tissue (Fig. 7e). We further characterized PPIL2 IHC expression in 34 breast ductal carcinoma cases, and defined an IHC score of ≤3 as being negative (Table 1). A *χ*^2^ analysis validated that PPIL2 expression was significantly correlated with breast cancer infiltration and lymphatic metastases (*P* < 0.05). In general, these results support our finding that PPIL2 acts as a crucial player in the suppression of metastasis in breast cancer.

**Table 1:** The expression of PPIL2 in breast cancer (cases)

Group	Cases	Negative cases	*χ* ^2^	*P* value
Infiltrating				
Yes	20	7(70.0%)	8.3300	0.004
No	14	0		
Lymphatic metastasis				
Yes	6	6(100.0%)	5.2041	0.023
No	28	8(28.6%)		

## Discussion

PPIL2 has been shown to play a pivotal role in Alzheimer’s disease^24^, but little is known about PPIL2-mediated biological events in cancer. Invasion and metastasis contribute to the poor outcome of patients with breast cancer^25,26^. Considering the role of PPIL2 on cell mobility, we focused on the function of PPIL2 in breast cancer metastasis. In this study, we demonstrated that PPIL2 is a novel regulator of breast cancer metastasis. PPIL2 contributed to F-actin arrangement in breast cancer cells. Downregulation of PPIL2 led to an increase in filopodia composed of numerous F-actin-filled protrusions at the front edge of MCF-7 cells, increasing migratory activity^27^. CsA treatment resulted in disorganization of actin fibers, as well as a decrease in actin fiber numbers in ZR-75-30 cells, similar to what is seen with CsA treatment in human osteosarcoma U2OS cells^28^. However, this effect was reversed by PPIL2 knockdown. In a human interactome analysis using three quantitative dimensions organized by stoichiometries and abundances, PPIL2 was found to interact with kinesin light chain 2 (KLC2) and kinesin light chain 3 (KLC3)^29^. KLC2 and KLC3 belong to a microtubule-associated force-producing protein family^30^. Whether PPIL2 regulates the function of microtubules is unclear. Furthermore, the actin cytoskeleton is not only required for cell motility, but it is also important in protein transport and surface remodeling^31,32^, which may suggest that PPIL2 plays an important role in proliferation, cell communication, and modulation of the tumor microenvironment in breast cancer^33,34^.

The U-box protein family had been defined as a family of special RING E3 ubiquitin ligases. The U-box domain likely functions similarly to the RING-finger domain in mediating ubiquitin conjugation of protein substrates^16,35,36^. Co-IP experiments showed that the U-box domain of PPIL2 was responsible for the interaction with SNAI1, as well as for stimulating ubiquitin transfer, similar to what was observed with a U-box-type ubiquitin ligase CHIP^37^. However, this observation differs from observations with another ubiquitin ligase, FBXO11. Jin et al. reported that the UBR motif of FBXO11 is dispensable for SNAI1 binding, while the F-box domain of FBXO11 is involved in enhancing polyubiquitination of SNAI1^38^. When SNAI1 levels were suppressed by PPIL2, lower levels of SNAI1 were bound to the promoter of the epithelial marker *CDH1*, resulting in an upregulation of *CDHI* expression. This might be the molecular mechanism connecting PPIL2 to EMT.

CsA is a commonly used immunosuppressant for organ transplant patients, with well-studied pharmacodynamics and safety profiles. PPIA is the main intracellular binding protein of CsA^7^. CsA represses the PPIase activity at Trp121 in the active site of PPIA^39^. Notably, PPIA failed to activate PRLr/Jak2 signaling when PPIase activity was reduced. This has been considered to be the mechanism for the inhibitory effect of CsA on breast cancer tumorigenesis and metastasis^10,40^. Surprisingly, PPIL2 is a unique member of the cyclophilin protein family, with low PPIase activity due to the presence of Tyrosine 389 rather than tryptophan at position 389 of the active site. Davis et al. showed that PPIL2 did not bind CsA in an isothermal calorimetry assay^22^. But our data demonstrate that CsA prolonged PPIL2 half-life and increased PPIL2 abundance in breast cancer. The role of PPIL2 in the CsA pathway may be more complicated in vivo. Current efforts are underway to clarify how CsA stabilizes PPIL2.

Although CsA was reported to induce EMT in gingival epithelium and renal tubular cells^41–43^, it repressed EMT in breast cancer. This indicates that the role of CsA in EMT is tissue dependent. The ubiquitinated SNAI1 accumulated in ZR-75-30 cells after exposure to CsA; however, PPIL2 silencing attenuated this effect (Fig. 5h). CsA also inhibited breast cancer migration and invasion, in a partially PPIL2-dependent way. These results indicate the predominant role of PPIL2 in the CsA pathway.

CsA takes part in complex signaling pathways in vivo, as it not only acts on the exogenous breast cancer cells, but it also affects various physiological activities of host cells in mouse breast cancer metastasis models. The changes in PPIL2 levels in mice injected with CsA were related to metastasis. However, the mechanism of action needs more exploration. CsA was observed to regulate the secretion of inflammatory factors, ultimately suppressing the immune response^44^ and achieving a change in the tumor microenvironment. The function of PPIL2 on the microenvironment has not been well studied. There are several nonimmunosuppressive cyclosporines^45–47^, however, their roles in breast cancer metastasis are still unclear. Thus, the mechanism by which CsA suppresses breast cancer metastasis in vivo deserves further investigation.

The efficacy of chemotherapy is limited due to drug resistance in breast cancer, therefore, it is important to overcome drug resistance to enable patient survival. Recently, a phase II study demonstrated that CsA plus oral docetaxel was an effective and safe treatment in anthracycline pretreated patients with advanced breast cancer^48^. In addition, CsA reversed resistance to the anticancer drugs gefitinib, mitoxantrone, and topotecan^49,50^. SNAI1 is a short-lived protein since it can be rapidly polyubiquitinated and degraded by the 26 S proteasome system. In stress conditions, such as those caused by chemotherapeutic drugs, SNAI1 protein turnover in more complicated^6^. Thus, studying the role of PPIL2 in drug resistance is of interest.

We provided a comprehensive model demonstrating that PPIL2 inhibits EMT and metastasis in breast cancer through cytoskeleton remodeling and by modifying SNAI1 ubiquitination. It was demonstrated that PPIL2 takes part in CsA-mediated inhibition of EMT in breast cancer. The potential use of CsA in breast cancer patients needs to be investigated in depth. Further investigations into PPIL2-mediated suppression of cancer metastasis may provide insight for extending the clinical use of CsA to breast cancer patients.

## Materials and methods

### Cell culture and plasmids

MCF-7 was cultured in Earles’s modified Eagle’s medium supplemented with 10% bovine calf serum and 10 μg/ml insulin. T47D cells were grown in Roswell Park Memorial Institute (RPMI) 1640 medium supplemented with 10% bovine calf serum and 10 μg/ml insulin. ZR-75-30 and 4T1/Luc cells were cultured as previously described^51^. MCF-10 was cultured in DMEM F12 (1:1) medium with horse serum (5%), insulin (10 μg/ml), epidermal growth factor (20 ng/ml), cholera toxin (100 ng/ml), and hydrocortisone (0.5 μg/ml).

### Plasmids and transfection

Flag × PPIL2 and HA × PPIL2 plasmids were constructed. The sequences of PPIL2 open-reading frame primers were as follows: PPIL2-F: 5′-CGGGATCCATGGGGAAGCGACAGCAC-3′, PPIL2-R: 5′-CGGAATTCCTAGTGGTCATCAGGCAGCC-3′. pcDNA3.1-3 × Flag, pcDNA3.1-3 × Flag-SNAI1, pEGFP-SNAI1, pGL3 vector, and pGL3-E-cadherin-Luc were obtained from our lab previously^52^. For plasmids of the Checkmate Mammalian Two-Hybrid System (Promega, Madison, WI, USA), PPIL2 and SNAI1 were subcloned into BamHI–EcoRV that cut pBIND and pACT, respectively. siPPIL2 targets to 3′UTR were purchased from GenePharma (GenePharma, Jiangsu, China) .The GFP-PPIL2-Ubox construct included only the U-box domain and GFP-PPIL2-PPI construct contained only the peptidylprolyl isomerases (PPI) domain. Two truncated SNAI1 plasmids GFP-SNAI1-NT (aa:1–151) and GFP-SNAI1-CT (aa:152–264) were constructed. Lipofectamine 2000 (Invitrogen, Auckland, New Zealand) was used for transfection according to the manufacturer’s specifications. HA-Ub, HA-K48-Ub, and HA-K63-Ub were kindly provided by Dr. Wenjun Liu (Chinese Academy of Science).

### Drugs and antibodies

Where indicated, the following drugs were used: MG132 (10 μM, Sigma, Saint Louis, MO, USA), cycloheximide (20 nM, Sigma, Saint Louis), CsA (2 μg/ml or 20 μg/ml, Sangon, Shanghai, China), and Leptomycin B (5 ng/ml, Beyotime, Shanghai, China). Rabbit anti-Flag, anti-GFP, anti-HA, and mouse anti-Flag antibodies were purchased from Sigma (Sigma, Saint Louis, MO, USA). Mouse anti-E-cadherin, anti-Cytokeratin 18, and rabbit anti-F-actin and anti-Vimentin antibody, rabbit anti-N-cadherin, and anti-SNAI1 antibodies were obtained from Abcam (Abcam, Cambridge, MA, USA). Rabbit anti-PPIL2 antibodies were purchased from Thermo Fisher Scientific (Thermo Fisher, Massachusetts, USA). Anti-fibronectin antibody was purchased from Wanleibio (Wanleibio, Shenyang, China).

### Western blot, Co-IP, chromatin immunoprecipitation, and GST pull-down assays

Western blot, Co-IP, chromatin immunoprecipitation, and GST pull-down assays were conducted as previously described^52,53^. GST-SNAI1 fusion proteins were expressed in Escherichia coli BL21 cells. GST pull-down assay was performed using a Pierce GST Protein Interaction Pull-Down Kit (Thermo-Pierce). The purified GST-SNAI1 fusion protein was immobilized on the PierceSpin Column according to the instruction. And then, PPIL2 was blotted from MCF-7 cell lysate.

### Reporter gene and quantitative RT-PCR

Reporter gene and quantitative RT-PCR were performed as described in our previous study^54^. The primers used for RT-PCR were as follows: qPPIL2-F: 5′-AAATACAAATGCCGAGAC-3′, qPPIL2-R: 5′-CTTCACAAACTGGTAGCG-3′, qCDH1-F: 5′-CTTCGGAGGAGAGCGGTG-3′, qCDH1-R: 5′-TCGTCCTCGCCGCC-3′, qCDH2-F: 5′-ATGAAACGCCGGGATA-3′, qCDH2-R: 5′-TCATCACCTCCACC-3′, GAPDH-F: 5′-TGA AGGTCGGAGTCAACGG-3′, and GAPDH-R: 5′-CCTGGAAGATGGTGATGGG-3′.

### Immunofluorescence and immunohistochemistry

Immunofluorescence and immunohistochemistry were performed as previously described^52^. To define the colocation of PPIL2 and SNAI1, the slides with fixed MCF-7 cells were incubated with both rabbit anti-PPIL2 and mouse anti-SNAI1 antibodies. For F-actin staining, the cells were incubated with rhodamine–phalloidin for 1 h at room temperature. The figures were obtained by fluorescence microscopy.

We collected five normal/pericarcinomatous human breast tissues, nine breast fibroadenoma tissues, and 34 ductal breast carcinoma tissues for immunohistochemical analysis from Qiqihar Medical University. All individuals who donated the tissues for this study gave their consent in written form. A IHC score of 0–8 (0 = negative or no expression; 1–3 = weak expression, <10%; 4–6 = moderate expression, between 10 and 50%; and 7–8 = high expression, >50%) was assigned to the expression level of PPIL2 and SNAI1 in breast tissues. Every section was scored according to the percentage of stained tumor cells and staining intensity.

### Transwell and scratch wound-healing assays

Before the transwell assays, cells were transfected with appropriate plasmids and selected for several days in the presence of G418. In the transwell assay, 1 × 10^5^ cells stably transfected with appropriate plasmids were plated on transwell chambers. Cell migration and invasion assays were performed in transwell chambers with or without Matrigel, which was used to simulate in vitro extracellular matrix. After being cultured for 24 h, the chambers were stained with crystal violet and then observed with a microscope. The invasive potential of MCF-7 and ZR-75-30 cells was studied in vitro by determining the number of cells that invaded through Matrigel-coated Transwell polycarbonate membrane. A total of 1 × 10^5^ cells that stably expressed appropriate plasmids were plated and cultured on a six-well plate for scratch wound healing. A wound was created by manually scraping the cell monolayer with an aseptic 200 -μl tip. Closing of the wounds was photographed at 0 and 24 h following wounding.

### Mice metastasis model

BALB/c mice (8-weeks old) were purchased from the Laboratory Animal Center of Dalian Medical University. To confirm the role of PPIL2 in metastasis in vivo, 18 mice were divided into three groups. Control BALB/c mice were injected with 100 μl of normal saline only (MOCK group). 4T1-pc3.1 group was injected with 1 × 10^9^ 4T1/Luc cells stably transfected with pcDNA3.1-3 × Flag vector into the tail vein. 4T1-PPIL2 group was injected with 1 × 10^9^ 4T1/Luc cells stably transfected with pcDNA3.1-3 × Flag-PPIL2. After 10 days, the animals were killed under an anesthesia and the lungs were removed surgically for the assay. To investigate the effect of CsA on PPIL2 in vivo, the mice were divided into the MOCK group (*n* = 5), the DMSO group (*n* = 5), and the CsA group (*n* = 8). The MOCK group was injected with 100 μl of normal saline into the tail vein only. The DMSO group and CsA group mice were injected with 1 × 10^9^ 4T1/Luc cells, respectively. On 5 days after injection, the mice in the CsA group were intraperitoneally injected with CsA (25 mg/kg/day) dissolved in 10% DMSO, while the DMSO group was injected with an equal volume of 10% DMSO. The MOCK group was treated with an equal volume of normal saline. On the 5th day, the mice were killed and their lungs were removed. The sizes and weights of all the mice lungs were recorded. A total of 50 mg of the lung tissue was ground with liquid nitrogen and homogenized in 200 μl of RIPA buffer A for western blotting. The rest of mouse lung tissues were used for subsequent hematoxylin and eosin staining.

### Statistical analysis

Data were presented as means ± SD($\overline x$ ± *s*) of at least three independent experiments. Statistical analyses were carried out with one-way analysis of variance with Bonferroni’s multiple-comparison test. Immunohistochemical data were determined by means of the two-sided *χ*^2^ test. Statistical significance was considered at *P* < 0.05.
